# Quo Vadis *Caenorhabditis elegans* Metabolomics—A Review of Current Methods and Applications to Explore Metabolism in the Nematode

**DOI:** 10.3390/metabo11050284

**Published:** 2021-04-29

**Authors:** Liesa Salzer, Michael Witting

**Affiliations:** 1 Research Unit Analytical BioGeoChemistry, Helmholtz Zentrum München, Ingolstädter Landstraße 1, 85764 Neuherberg, Germany; liesa.salzer@helmholtz-muenchen.de; 2 Metabolomics and Proteomics Core, Helmholtz Zentrum München, Ingolstädter Landstraße 1, 85764 Neuherberg, Germany; 3 Chair of Analytical Food Chemistry, TUM School of Life Sciences, Technical University of Munich, Maximus-von-Imhof-Forum 2, 85354 Freising, Germany

**Keywords:** *Caenorhabditis elegans*, metabolomics, lipidomics, mass-spectrometry, NMR

## Abstract

Metabolomics and lipidomics recently gained interest in the model organism *Caenorhabditis elegans* (*C. elegans*). The fast development, easy cultivation and existing forward and reverse genetic tools make the small nematode an ideal organism for metabolic investigations in development, aging, different disease models, infection, or toxicology research. The conducted type of analysis is strongly depending on the biological question and requires different analytical approaches. Metabolomic analyses in *C. elegans* have been performed using nuclear magnetic resonance (NMR) spectroscopy, direct infusion mass spectrometry (DI-MS), gas-chromatography mass spectrometry (GC-MS) and liquid chromatography mass spectrometry (LC-MS) or combinations of them. In this review we provide general information on the employed techniques and their advantages and disadvantages in regard to *C. elegans* metabolomics. Additionally, we reviewed different fields of application, e.g., longevity, starvation, aging, development or metabolism of secondary metabolites such as ascarosides or maradolipids. We also summarised applied bioinformatic tools that recently have been used for the evaluation of metabolomics or lipidomics data from *C. elegans*. Lastly, we curated metabolites and lipids from the reviewed literature, enabling a prototypic collection which serves as basis for a future *C. elegans* specific metabolome database.

## 1. Introduction

### 1.1. Caenorhabditis elegans—A Versatile Model Organism

The small nematode *Caenorhabditis elegans* (*C. elegans*) is a frequently used model organism in biomedical research. It was introduced in 1973 by Sidney Brenner as promising model for developmental genetics [1]. The nematode is of microscopic nature with adults having an approximate length of 1 mm and a diameter of 80 µm. *C. elegans* can be found all over the world in moderate climates, mostly in garden soil and compost, but it has been isolated also from snails, woodlice, and other invertebrates [2]. It shows a bacterivorous feeding on bacteria growing on rotting biomaterial.

The main benefits and reasons of using *C. elegans* as a (genetic) model are its simplicity, and speed of cultivation. Under laboratory conditions it has a short generation time (3 days from fertilized egg to sexually mature adult), and a short lifespan of about 2–3 weeks at 20 °C. Cultivation in the laboratory takes place on a solid support such as agar plates or in liquid by feeding with bacteria, normally with *Escherichia coli* (*E. coli*), which is cheap, easy and can be scaled up to high-throughput screening approaches [3]. *C. elegans* has two sexes and predominantly exists as self-fertilizing hermaphrodites, typically only 0.1% appear to be males. Hermaphroditic reproduction also leads to low genetic variation within a culture. Another characteristic is the invariant development, leading to exactly the same number of somatic cells in each individual (959 and 1031 in hermaphrodites and males respectively). *C. elegans* develops through four larval stages (L1–L4) into reproductive adults after hatching. When no food is available or populations are overcrowded, normal development is arrested and L1 larvae enter an alternative life cycle and become so-called “dauer” larvae (“dauer” german for enduring). The cuticles of dauer larvae are highly stable and protect the worm against environmental factors, such as desiccation. In dauer-state, worms can survive several months without food. As soon as food is available again normal development continues through the L4 stage into reproductive adults [4].

About 60–80% of the nematode genes are homologues to human genes [5]. Therefore, investigations on *C. elegans* can have high relevance to studies of human health and disease. Different forward and reverse genetic tools are available in *C. elegans*. RNA interference (RNAi) has become one of the most common used methods for systematic gene inhibition in *C. elegans* [6,7,8]. RNAi inhibits gene activity by introducing double-stranded RNA (dsRNA) with a sequence specific for the target gene. This leads to degradation of the homologous messenger RNA (mRNA) [5,9,10]. Also, clustered regularly interspersed short palindromic repeats (CRISPR)–Cas9 has been applied in *C. elegans* for genome engineering, which has been reviewed elsewhere [11,12,13,14,15,16,17,18].

One of the most recent additions to the *C. elegans* toolbox are metabolomics and lipidomics, enabling new and deeper investigations into the metabolism of the nematode. The combination of a genetically tractable model organism such as *C. elegans* with the functional readout of metabolomics and/or lipidomics holds great promise to advance our knowledge on metabolism and metabolic regulation. 

### 1.2. Metabolomics and Lipidomics—Systematic Measurements of Metabolites and Lipids

Metabolomics and lipidomics are defined as the systematic measurement and quantification of metabolites or lipids in a given system. They probably represent the most complex of all “-omics” approaches due to the large chemical complexity and concentration range underlying the metabolome and lipidome. Metabolism is of great interest, since it is closest to the observed phenotype and typically is one of the first things to react upon a stimulus. Metabolite concentrations are directly linked to biochemical activity, and many biological processes depend on metabolism [19,20]. Metabolomics also enables the investigation of interactions of an organism with its environment or between organisms. The goal of metabolomics is to describe all metabolites in a biological system in a defined state [21]. 

One can distinguish between targeted and non-targeted metabolomics. In targeted approaches, a predefined set of metabolites is studied. Since these targets are known beforehand, methods are optimized on existing chemical reference standards. Therefore, their analysis is highly precise, accurate and often allows absolute quantification. Metabolites investigated in targeted analysis typically belong to a single class of compounds or a few related compound classes or biochemical pathways, whereby the use of a single analytical technique might be sufficient. 

However, targeted metabolomics requires a specific hypothesis on changes in the metabolism, which needs to be tested. In contrast, non-targeted metabolomics characterizes a large number of metabolites by their simultaneous measurement without prior selection and free of any hypothesis. Therefore, the data of non-targeted metabolomic approaches is much more complex and often requires more elaborate statistical and bioinformatic evaluation. A major issue in non-targeted metabolomics in general is the lack of comprehensive measurements of the whole metabolome by a single technique due to its large complexity. At the current state it is not possible to measure the whole metabolome of an organism in a single experiment. Beside several known compounds a large part of the metabolome has not even been identified yet (“metabolic dark matter”) [22,23]. To deal with the large chemical complexity, complementary analytical platforms are required. Metabolomics typically employs high end analytical methods, such as mass spectrometry (MS)—either direct infusion (DI) or hyphenated to different types of separation (gas chromatography (GC), liquid chromatography (LC), capillary electrophoresis (CE) or ion mobility (IM))—or nuclear magnetic resonance spectroscopy (NMR).

*C. elegans* possess a complex metabolome and several new molecules are described on a routine basis, e.g., novel ascarosides, dafachronic acids or others [24,25,26,27,28,29,30]. Different types of analytical methods have been used to analyze the *C. elegans* metabolome and lipidome. 

## 2. Analytical Methods for *C. elegans* Metabolomics

The following paragraphs give an overview on different extraction and analysis methods that have been used to analyze the *C. elegans* metabolome and lipidome. This overview shall help scientists new to the field to search for their most fitting combination of extraction and measurement methods for their specific application.

### 2.1. Extraction Methods

The first step towards analysis of the metabolome and lipidome is the extraction of compounds of interest from the nematodes. In case of non-targeted analysis extraction of as many substances as possible is required. It has to be mentioned that a truly non-targeted extraction does not exist, since different solvent systems will always favor specific metabolites and metabolite classes over others. Furthermore, extraction depends on the biological question and on the analytical method that shall be used for analysis. In order to analyze the metabolome as accurately as possible, it is also necessary to avoid degradation of the metabolites during extraction. Therefore, extractions shall be carried out at the lowest possible temperatures. Extraction of metabolites from *C. elegans* is challenging since the nematode possess a hard cuticle, which first needs to be broken before extraction. However, the type of tissue disruption seems to be a less critical step in sample preparation as shown by Geier et al. who investigated different disruption techniques in *C. elegans* including manual grinding, homogenization and different grinding media in tissue mills [2]. However, care must be taken to avoid excessive heating of samples e.g., when using bead beating systems, since it may cause sample degradation. In contrast, the extraction procedure itself is much more important. Different extraction methods have been applied to *C. elegans*. Most commonly (cold) methanol, ethanol, and chloroform, either individually or in combination (in diverse rations) have been applied [29,31,32,33,34]. For lipid extractions on the other hand, protocols from Folch and Bligh & Dyer are often used [35,36]. Thereby, lipids are extracted and separated into the hydrophobic chloroform phase and other compounds into the methanol/water phase. Since chloroform is suspected to cause cancer its reduction to minimal amounts or complete replacement is desirable. Matyash et al. developed an extraction based on methyl-tert-butyl-ether (MTBE) using also *C. elegans* as a model system. Extraction yields were similar to other lipid extraction methods. A further advantage is that MTBE forms the upper phase in a two-phase extraction with water, making recovery of lipids easier and even automatable [37]. 

Analysis of specific metabolites or lipids often requires their enrichment using further sample preparation steps. Thin layer chromatography (TLC) is an approach used either in analytical purposes to determine lipid composition, or as additional step for preparation of specific substance classes. In TLC major lipid classes as glycerophospholipids, ceramides, glycosphingolipids, fatty acids, or sterols can be separated and quantified. Chromatographic resolution may be enhanced through its extension to two-dimensional TLC using a second solvent system [30,38].

Another challenge in metabolomics is the detection of low abundant compounds, since the detection limit of the analysis is restricted depending on the sensitivity of the employed technique. Metabolites are enriched, e.g., by using solid phase extractions (SPE) or preparative chromatography [22,25,29]. For example, Hänel et al. used fractionation based on aminopropyl and weak anion exchange SPE of lipid extracts in order to enrich sphingolipids from *C. elegans* [39]. 

### 2.2. Nuclear Magnetic Resonance (NMR)

Nuclear magnetic resonance (NMR) spectroscopy is based on the interaction between the magnetic moment of atomic nuclei and a magnetic field. Only nuclei that have a nuclear spin do have a magnetic moment and thus are NMR active. In an NMR experiment the free induction decay (FID) is obtained, which is the magnetic resonance response in a unit of time. Via fourier-transformation (FT) the NMR spectra are generated, consisting of signal intensities of NMR active compounds depending on their resonance frequency. The most important nuclei at the analysis of biomolecules are ^1^H, ^13^C, ^15^N, ^19^F and ^31^P.

In metabolomics mostly one dimensional ^1^H NMR spectroscopy is used, since it is fast and very convenient for universal detection of organic compounds. NMR is quantitative and nondestructive with minimal sample preparation and interference, resulting thus to lowest analytical variation compared to other techniques [40]. Its high precision allows the detection of even small changes in metabolite abundances. NMR delivers qualitative (structure) and quantitative information in a single run. Its strengths are particularly evident in substances that are difficult to ionize in MS or require derivatization. However, a major disadvantage of NMR compared to MS is the lower sensitivity. Therefore, only few tens of high abundant compounds are covered by 1D-^1^H NMR [41]. 

In order to increase metabolite coverage in non-targeted metabolomics, 1D-^1^H NMR is often combined with other analytical platforms such as DI-MS, GC-FID, GC-MS, or LC-MS [42,43,44,45]. 

In *C. elegans* metabolomics 1D-^1^H NMR has been used to investigate changes is central carbon metabolites such as amino acids, organic acids, choline, sugars, nucleotides or cofactors [42,45,46,47,48]. A limitation in 1D-^1^H NMR is overlapping signals, resulting in ambiguity in metabolite identification and quantification. Extension to 2D-NMR is often used to confirm elucidated structures and thus improve identification rates. 2D-NMR approaches such as ^1^H-^1^H correlated spectroscopy (COSY), ^1^H-^1^H total correlation spectroscopy (TOCSY), and ^1^H-^13^C heteronuclear single quantum coherence (HSQC) have been applied in *C. elegans* metabolomics [30,49,50,51]. 

Another approach to enhance NMR structural resolution is performing ^13^C-Heteronuclear Multidimensional NMR (HMN) instead of 1D-^1^H NMR. Due to the low natural abundance of ^13^C, ^13^C HMN suffers from low sensitivity. Using in vivo labeling with ^13^C can increase sensitivity of ^13^C HMN. ^13^C-labeling of *C. elegans* is possible by first feeding ^13^C-glucose to *E*. *coli*, followed by feeding labeled bacteria to the nematodes. Such labeling and ^13^C HMN was applied to metabotyping of *C. elegans* mutants by An et al. [52]. Labeling increased sensitivity two orders of magnitude compared to unlabeled samples. It was also possible to perform high resolution two-dimensional HSQC and three-dimensional HCCH-TOCSY NMR experiments [53]. This enabled the detection of much more metabolites than in one-dimensional NMR metabolomics. The major drawback of performing 2D-^13^C HSQC experiments using ^13^C-labeled metabolites is additional structures arising from ^13^C/^13^C couplings, which are non-existent at natural ^13^C abundance. These additional structures can reduce the possible sensitivity gain of ^13^C-labeling and increase chances of peak overlap. Therefore, Geier et al. investigated different HSQC pulse programs for fully ^13^C-labeled tissue extracts from *C. elegans*. They found at constant time HSQC (ct-HSQC) improved peak shape and better peak detection of metabolites, which enabled matching of 300 records from Human Metabolome Database (HMDB) [54,55,56,57,58].

Another promising approach developed using *C. elegans* is differential analysis by 2D-NMR spectroscopy (DANS). DANS is a method based on overlaying and subtracting 2D-NMR spectra from two different conditions. Therefore, detailed structural information of differentially regulated compounds can be directly extracted, even for minor components in complex matrices [26,59]. 

To circumvent extraction, nematodes can be directly analyzed using high-resolution magic-angle spinning (HR-MAS) NMR spectroscopy [60,61]. At HR-MAS the sample is spinned at a distinct angle to the magnetic field to overcome magnetic field heterogeneities within solid samples that are responsible for broadening of resonance lines. The main advantage over other platforms such as in-solution NMR, or MS is that no metabolite extraction is required and *C. elegans* can be directly filled into a HR-MAS NMR rotor. Because of the general insensitivity of NMR, around 1000 nematodes are required for a standard HR-MAS analysis. Wong et al. introduced high-resolution magic-angle coil spinning (HR-MACS) combined with a ^1^H NMR microprobe for the metabolic phenotyping of low number of *C. elegans* [62]. Sensitivity is improved by miniaturizing the rf receiver coil (µcoil). Moreover, an inductively coupled µcoil resonator is spinning together with the sample, which suppresses magnetic susceptibility broadening from both the µcoil and the sample. HR-MACS NMR allowed metabolic profiling from small numbers of nematodes ranging from 10 to 100. It was even possible to acquire a NMR spectrum of a single worm on a 1000 MHz (23.5 T) spectrometer [63]. 

### 2.3. Mass Spectrometry (MS)

Mass Spectrometry (MS) is beside NMR one of the major analytical technologies used in metabolomics. A mass spectrometer is an instrument which generates ions and separates them according to their respective mass-to-charge ratio (m/z). The ions are separated due to different physical approaches, depending on the type of mass spectrometer being used. In MS m/z ratios of ions are measured together with their corresponding intensities, combining both qualitative (m/z) and quantitative (intensity) information. However, metabolites with same molecular formula have the same mass and therefore the same m/z value (isomers, e.g., Leucine and Isoleucine, C_6_H_13_NO_2_). Likewise, several sum formulae might have very similar but not identical masses and m/z values (isobars, e.g., [M+Na]^+^ adduct of PC(34:1) and [M+H]^+^ adduct of PC(36:4)). Such compounds may be further analyzed using tandem MS (sometimes referred to as MS^2^ or MS/MS) experiments. Here, the ions of interest are fragmented, mostly using collision induced dissociation (CID), and fragment ion m/z values are analyzed yielding information on potential substructures. In non-targeted metabolomics, mostly data dependent acquisition (DDA) is used to generate and acquire MS/MS data. In DDA the most intense ions, meeting certain user defined thresholds, are selected and fragmented. Another approach that is increasingly used in non-targeted metabolomics is data independent acquisition (DIA), such as Sequential Window Acquisition of all Theoretical fragment ion spectra (SWATH) MS or All ions. Benefit of DIA is that every precursor ion will be fragmented, allowing retrospective data analysis without the need of reacquisition.

MS is either employed without or with prior chromatographic or electrophoretic separation. The employed ionization source depends on the upfront sample introduction system, e.g., electron ionization (EI) or chemical ionization (CI) for GC, electrospray ionization (ESI) and atmospheric-pressure chemical ionization (APCI) for direct infusion, LC or CE. 

In direct infusion (DI) MS the sample is directly injected into the ionization source without prior separation. In order to differentiate between isobaric structures, the use of high-resolution (HR) MS, such as time-of-flight (TOF) MS, Fourier-transform ion cyclotron resonance (FT-ICR) MS, or Orbitrap MS, is a prerequisite at DI-MS.

Separation is used to reduce ion suppression and separate isomeric and isobaric structures, which cannot be separated by MS or MS/MS alone. Furthermore, additional information about physicochemical properties of detected metabolites based on the respective separation characteristics is provided.

GC has been adopted early for the analysis of endogenous metabolites, even before the term metabolomics existed, e.g., Pauling et al. used GC for the analysis of urine and breath [64]. GC-MS offers highly efficient and highly reproducible separation of volatile metabolites or metabolites that can be made volatile by derivatization. GC is typically combined with EI, which produces highly reproducible fragmentation rich spectra. Therefore, GC-MS analysis allows ready dereplication of known and previously measured metabolites with many spectral libraries available. However, if unknown substances are analyzed CI might be preferred, since the parent ion is mostly preserved, as for instance shown by Jaeger et al., who were using GC-APCI-MS for metabotyping in *C. elegans* [65].

If substances are not volatile or can be made volatile by derivatization, LC-MS might be used for analysis. While selection of GC stationary phases is rather limited, LC offers a large selection of different column chemistries to optimize metabolite separations. In LC metabolites are retained depending on the stationary and mobile phase and their physicochemical properties. Today, Ultra-high-performance liquid chromatography (UPLC), using particle sizes of below 2 µm, is widely used in non-targeted metabolomics, due to fast and efficient separation [66]. 

Despite the large number of possible separation chemistry available, reversed-phase (RP) columns are used most. They allow the separation of mid- to non-polar metabolites and lipids. However, very polar metabolites are not retained on RPLC columns. Therefore, hydrophilic interaction liquid chromatography (HILIC) is frequently used. HILIC columns are either silica or derivatized silica columns [34,67]. However, polar, charged metabolites often show bad peak shape in LC. Capillary zone electrophoresis (CZE), normally referred to as CE, coupled to MS is another rising powerful analytical technique in metabolomics [68]. In CE charged molecules migrate in a liquid background electrolyte along an electrical field. Separation occurs due to different velocities of the metabolites, depending on their mobility and the electric field strength. CE requires only a small sample amount, only a few nl per injection. It is well suited for highly polar and charged metabolites but has not been applied to *C. elegans* so far. Its usability on metabolomics in general has already been reviewed elsewhere [69].

Peak capacity, spectral clarity and fragmentation specificity of metabolomics LC-MS data can be further increased by adding ion mobility (IM) separation as a second separation dimension post ionization. Moreover, IM provides an additional parameter that may be useful for metabolite identification—the collision cross-section (CSS) value. At IM separation takes place in the gas phase due to different ion mobilities, that depend on charge, shape and size of the metabolites. Its applicability on metabolomics and lipidomics in general have already been confirmed [70]. 

Most of the mentioned analytical techniques have also been used for the analysis of the *C. elegans* metabolome. Table 1. summarizes the analytical methods used in *C. elegans* metabolomics and lipidomics, including their advantages and disadvantages. MS has already been demonstrated to be an effective tool for metabolomic profiling in *C. elegans*. For instance, DI-MS is frequently used for the analysis of the *C. elegans* lipidome [30,71]. Identification of lipids is thereby accomplished on their accurate mass and/or MS/MS characterization. GC-MS has been used for metabolomics in *C. elegans* to identify fatty acids, amino acids, or organic acids [31,42,43,50,59,65,72,73,74,75]. Not only coupled to MS, but also GC using a flame ionization detector (FID) has been used as a complementary analytical platform in *C. elegans* to determine the fatty acid composition [42,43,45]. UPLC has also frequently been applied to separate lipids and metabolites of *C. elegans* prior to mass spectrometric analysis [44,51,73,76]. RPLC columns are used for lipids and mid- to non-polar metabolites. Lipidomic studies were mostly accomplished by C18 [31,38,43,76,77,78] but also C8 stationary phases [42]. For polar metabolites on the other hand HILIC columns were predominantly used [34,67]. Moreover, van Assche et al. used aqueous normal phase (ANP) columns for the separation of polar metabolites in *C. elegans* [79]. Besides various column selectivity, separation is also influenced by additives, as shown by Wang et al. who used ion-pairing reversed phase in negative ion ESI mode, where tributylamine was added to the aqueous mobile phase [74]. Analytical platforms have been used individually and in combination used to enhance the coverage of the metabolome. 

### 2.4. Bioinformatic Tools for the Analysis of the C. elegans Metabolome/Lipidome

Beside the actual chemical analysis of the metabolome and/or lipidome, extensive bioinformatic and statistical analysis is required to interpret large data sets produced using the aforementioned analytical approaches. Evaluations on all types of data, either NMR or MS based, were most commonly accomplished using multivariate data analysis methods. Multivariate methods can identify relationship patterns between metabolites by clustering and correlation analysis. Widely used chemometric methods such as principal component analysis (PCA), hierarchical cluster analysis (HCA), or orthogonal projection to latent structure with discriminant analysis (OPLS-DA) have been applied [88,101,102]. 

Moreover, metabolite and spectral databases have become indispensable for the analysis of metabolomics data and the identification of metabolites. Through searching against in-house and public databases, metabolites can be annotated and identified at different levels according to the Metabolomics Standard Initiative [103]. Most commonly HMDB (http://www.hmdb.ca) [55], METLIN (http://metlin.scripps.edu/index.php) [104], KEGG (http://www.genome.jp/kegg/), BMRB (http://www.bmrb.wisc.edu) [105], or LipidMaps (http://www.lipidmaps.org) [106] are used for this purpose. However, they are only partially useful for *C. elegans* metabolomics. At the current stage, no *C. elegans* metabolome specific database exists. The Small Molecule Identifier Database (SMID DB, http://www.smid-db.org/) is storing information on *C. elegans* and related species-specific secondary metabolites, such as ascarosides and recently added MS^2^ spectra and retention times [22]. Still, the exact number of metabolites in the *C. elegans* metabolome remains elusive. 

Genome-scale metabolic models (GSMs) are aiming to close this knowledge gap and to deliver computer readable mathematical models of the nematode’s metabolism. For *C. elegans* four different models have been published. Path2Models convert metabolic pathways from KEGG into SBML models, which were then gap-filled [107]. The model reconstructed by Gebauer et al. contains 1921 reactions and 2357 metabolic compounds of *C. elegans* [108]. Moreover, Yilmaz et al. created a model including 1985 reactions and 1718 compounds based on SACURE curative approach [109]. The fourth model CeCon was developed with the PathwayTools software [80]. At last, the WormJam model has been created with the existing metabolic reconstructions of *C. elegans* have been merged into a single consensus model. WormJam is freely available and continuously improved to cover different aspects of *C. elegans* metabolism [110,111,112]. Furthermore, first steps towards the development of tissue and cell type specific models of *C. elegans* have been made [14]. Since WormJam is curated by using only metabolites relevant to *C. elegans*, it may be useful to accelerate annotation of metabolomics data and avoid over annotation.

Additionally, pathway and network analysis are increasingly used to evaluate metabolomics experiments. Pathways found in *C. elegans* and other organisms were documented and visualized for instance in the PathBank database (http://www.pathbank.org) [113]. Pathway analysis uses prior biological knowledge to analyze metabolic patterns. Network analysis on the other hand uses correlation of metabolites to build metabolic networks that outline relationships between the metabolites. The basis of the network is therefore the experimental data itself [114]. Krumsiek et al. developed gaussian graphical model (GGM), which is based on partial correlation between metabolites, and has also been applied on *C. elegans* metabolomics data [43,115]. Compared to common correlation networks, GGM’s enable differentiation of direct and indirect interactions between metabolites. Lastly, molecular networking based on analysis on MS/MS fragmentation patterns have been used to evaluate *C. elegans* metabolomics data [22,92].

Flux balance analysis (FBA) is a mathematical method that analyzes the flow of metabolites in a metabolic network and have also been recently used for investigating *C. elegans* metabolism [31,67,116]. Using FBA, it is possible to determine pathways that are influenced if concentrations of a certain metabolite changes. FBA can integrate metabolomics data to constraint reaction boundaries, e.g., Yilmaz et al. used FBA to analyze metabolites from tissue specific pathways in *C. elegans* [117]. 

## 3. Applications of *C. elegans* Metabolomics

The previously mentioned metabolomics techniques are used to identify differentially regulated metabolites between different states that are involved in relevant biological pathways or that are specific for the respective state (biomarker). Frequently, studies on development, ageing, longevity, pheromones, lipid metabolism and regulation, effect of genetic perturbations, exposure to different chemicals and effect of different diets were investigated in the model organism *C. elegans* using metabolomics and lipidomics.

### 3.1. Development, Ageing and Longevity

Different studies investigated the relationship between ageing, longevity and metabolism in *C. elegans*. There are several mutants of *C. elegans* that increase its lifespan [118]. Frequently, non-targeted metabolomics has been used to analyze how the metabolism of those long-lived worms changed in comparison to controls with normal lifespan.

For instance knockout of genes involved in the Insulin/Insulin-Like Signaling (IIS) pathway tend to increase longevity [118]. One of the most studied mutants in *C. elegans* is *daf-2*. *daf-2* encodes for the orthologue of the insulin/insulin-like growth factor (IGF) receptor.

An altered amino acid profile and changes in the carbohydrate metabolism had been found to be a key feature in *daf-2* mutants. A common trend for different alleles of *daf-2* and *daf-28* (which disrupts a *daf-2* ligand) is the increase of branched chain amino acids (BCAA) leucine, isoleucine and valine [43,83] For example, Fuchs et al. used NMR based metabolomics to study three different alleles of *daf-2(e1370, m21 and m596)* as well as *daf-28(sa191)* and *ife-2(ok306)* [47]. Martin et al. similarly compared *daf-2(e1370)* and *daf-2(e1370)*; *daf-16*(m26) mutants [46]. However, BCAA levels drop at later stages as shown by Davies et al. who were performing a time course analysis of metabolites across age using NMR. Another interesting trend was seen for trehalose. This metabolite increases strongly throughout aging in *daf-2(m41)* mutants. Trehalose has been previously shown to increase the lifespan of wildtype worms but did not further extend the lifespan of *daf-2* mutant worms [81]. 

The lifespan of *daf-2* mutants is even further enhanced by additional knock-outs, as for example *pept-1(lg601)*, which encodes for an intestinal di- and tripeptide transporter [46] or prohibitin deficient *daf-2(e1370)* mutants shown by Lourenço et al. However, prohibitin deficiency shortens the lifespan of wild type nematodes. Fatty acid composition, and amino acid and carbohydrate metabolism, analyzed by NMR, was more deeply affected by prohibitin depletion in wild type nematodes compared to *daf-2* mutants. GC-FID analyses showed that prohibitin depletion of *daf-2* results in changes of polyunsaturated fatty acid contents, which has also found to be a key feature of longevity [45]. Also, lipid analysis showed an increase in triacylglycerols, especially containing branched and monounsaturated fatty acids in *daf-2* mutants [81].

Moreover, Prasain et al. performed lipidomic analysis of *daf-1* and *daf-2* mutants using a workflow called MSMS^ALL^. They identified changes in several phospholipid classes as well as di- and triacylglyerols. *daf-2* mutants are described to show a fat phenotype with higher number of lipid droplets. This is mirrored by the lipidomic analysis showing that *daf-2(e1370)* have higher levels of triacylglycerols compared to wildtype [119]. 

Metabolomics is able to produce a static snapshot of the current metabolic state. However, in most cases it remains elusive how this state was reached in regard to which pathway was active. The use of isotopic tracers can help to capture the dynamic nature of metabolism. Tracer analysis in *C. elegans* represents a complicated task since no axenic medium is available and always co-metabolism of tracers between *E*. *coli* and *C. elegans* would be determined. Perez and van Gilst developed an interesting approach that allows to follow lipogenesis in *C. elegans* based on the feeding of a mixture of isotopically non-labeled or labeled *E*. *coli*. The *E*. *coli* food source was grown in either non- or fully-labeled growth medium. *C. elegans* was then fed with a 50/50 mixture of non-labeled and labeled *E*. *coli*. Fatty acids from *C. elegans* have been analyzed with GC-MS. Fatty acids directly derived from *E*. *coli* are either 100% non-labeled or labeled, while all fatty acids that are produced by *C. elegans* show a mixed labeling pattern. Based on the degree of labeling it can be determined if complete *de novo* synthesis or elongation has been performed. This method was applied to different alleles of *daf-2*: m577, e1368, m596, e1371, e1370 and m41. Interestingly, not all alleles showed the same extent in changes of *de novo* fatty acid synthesis, with highest levels found in *daf-2(1370)* and *daf-2(m41)* [89].

Recently, a ring trial comparing the WT and the *daf-2(e1370)* lipidome has been performed in order to investigate inter-laboratory reproducibility. Varying overlap of differentially regulated lipids from different laboratories have been observed. Main cause for these differences appeared to be the fold changes between both variants and the prerequisite of lipids being present in at least 80% per biological group. These ring-trials highlight the variance in lipid profiles that arises due to the small differences in cultivation between different laboratories. However, the most important markers for *daf-2*, as for example higher number of TG’s or decrease in PC (20:5/20:5), have been found in the majority of laboratories [120]. 

Beside *daf-2* different other genetic perturbations lead to increased lifespan. *eat-2* and *slcf-1* mutants, which are often used as genetic models for dietary restriction, also extend lifespan of the nematode. Both mutants show lower phosphocholine levels and therefore correlate with longevity. Additional mutation of *daf-18* suppressed the longevity phenotype and increased phosphocholine and choline kinase levels. Older animals having higher phosphocholine levels seem to have an increased choline kinase (*ckb-2*) expression, probably in adaptation to stress. Moreover, it was found that inactivation of *ckb-2* shortened the lifetime of the nematodes [61].

Shared changes in metabolic pathways between IIS (*daf-2*) and DR (*eat-2*) mutants that promote longevity in *C. elegans* were investigated by Gao et al. using metabolomics and transcriptomics. In both mutants an increase of glycerolipid intermediates, purine degradation intermediates and AMP have been observed. On the other hand, amino acid levels and certain fatty acids decreased. Shared longevity signatures in the transcriptome and metabolome were an increase in amino acid metabolism, probably due to lower protein synthesis, and up-regulation of purine metabolism [34]. In a targeted GC-MS approach it was shown that also N-acylethanolamines are involved in lifetime extension since they are decreased at dietary restricted, long lived worms [72]. 

Dauer larvae live about eight times longer than WT worms, making them also suitable for metabolomic investigations in terms of longevity. The metabolic profiles of dauer larvae were significantly different from WT worms but similar to other long-lived mutants. Consistent to other studies the metabolic signature of longevity contains metabolites of distinct pathways as carbohydrate, amino acid, and choline metabolism [47,83,121].

It was reported that high levels of SIR-2.1 (sirtuins, which are NAD+ dependent protein deacetylases) extend the lifespan from *C. elegans* up to 50%. Differential ^13^C HMN metabolomics of *sir-2*.*1* mutants and WT showed especially differences in branched chained amino acids, triacylglycerol, carnitine and acetyl-CoA, which were elevated in WT. Lactate, alanine, glutamate, fatty acids and AMP on the other hand were upregulated in *sir2*.*1* mutants. These results point to an increase in glycolysis, nitrogen catabolism and initial lipolysis in *sir2*.*1* [52].

On the contrary, short-lived worms, such as in mitochondrial mutants (*mev-1*(kn1)), showed upset TCA cycle balance, elevated lactic acid fermentation and increased amino acid catabolism, which has been found by Jaeger et al. using GC-EI-MS and GC-APCI-MS [65].

Ablation of germline stem cells (GSCs) leads to infertility and also extends lifespan. The germline-less *glp-1* mutants show an altered lipid metabolism compared to wildtype worms. Moreover, many age-related metabolites including increased levels of pyrimidine and purine metabolism intermediates and decreased concentration of citric acid cycle metabolites were differentially regulated in *glp-1* mutants. However, *glp-1* mutant worms exhibit the same tendency of metabolic changes as WT during aging, which was measured by both NMR and UPLC-MS. Some age-related metabolites in are dysregulated in the *glp-1* mutants (e.g., valine, GSSG, leucine, malate, serine), although some were not (e.g., cystathionine, glycine, arginine, trehalose). These results indicate that not all aspects of aging are present in long-lived *glp-1* mutants [44]. Future meta-analysis of several aging related studies may pinpoint towards shared principles of metabolism between the different longevity regimes.

Since elevated trehalose have frequently been observed at long-lived mutants, researchers tested if trehalose supplementation increases lifespan of WT worms. Middle aged WT worms showed prolonged lifetime but not early-stage adulthood nematodes. Moreover, at aged worms (10 days adults vs young adults (YA)) levels of glutathione were reduced, while oxidized glutathione was increased. Taurine and hypotaurine, which are antioxidants, were declined with age. Phosphocholine and trehalose have higher concentration with age and decreased levels of purine intermediates were observed. Concentrations of intermediates of pyrimidine and TCA cycle were found to be variable [81].

Pontoizeau et al. investigated aging effects on the metabolism by comparing metabolites of YA and adult (7 days adults) worms by means of HR-MAS NMR. Metabolites that increased with age were saturated and unsaturated lipids, glycerophosphocholines, phosphocholine, glutamine and glycine. Metabolites that decreased were a range of amino acids (alanine, arginine, isoleucine, leucine, lysine, phenylalanine, tyrosine, valine, glutamate), acetate, and lactate, glycerol, formate and cystathionine [61]. 

Age-related metabolites have also been found in adult worms at different time points by. Hastings et al. Levels of some amino acids (serine, threonine, leucine, lysine, glutamine/glutamic acid, methionine, tryptophan, arginine, homoserine, cystathionine), and nucleotides (guanosine, cytidine, uridine, GMP) decreased over time while some other metabolites (betaine, carnitine, leucic acid, pantothenate, kynurenic acid, xanthurenic acid) and degradation products of both nucleotides (hypoxanthine, xanthosine, allantoin) increased. An interesting aspect has been the integration of metabolomics data with FBA using an approach called MetaboFBA. Differences in fluxes using standard FBA compared to MetaboFBA have been observed, proving the importance of including metabolomics data in silico modeling approaches [67].

Metabolic changes between embryos, larval stages L1–L4, and adults have also been investigated. The lowest abundance of most fatty acids was found during larval development and levels increased at the reproductive phase of adults. Most, but not all, amino acids had highest levels at L3 stage and early adulthood and deceased throughout adult phase. Asparagine on the contrary was high in worm eggs and larval stages, but then declined throughout development. Some particular glycerophospholipids, such as many phosphatidylcholines (PCs) and phosphatidylethanolamines (PEs), were present in higher abundance during the larval stage and early adulthood (day 1) and lowered during adult lifespan. Lysophosphatidylethanolamine (LPEs) levels show similar trends, while some cardiolipins (CLs) had low abundance in L2 stage and increased during early adulthood. Other phospholipids such as phosphatidylglycerol (PG) and a sphingomyelin (SM) species showed an opposite pattern; they were less abundant during the larval and early adult stages and accumulated at a later stage of life (day 10) [84].

Adult lifespan and larval development are regulated by *daf-12*, a nuclear hormone receptor that functions as a ligand-dependent switch. 2D-NMR and DANS of WT and *daf-12* mutants combined with activity guided fractionation has been used to identify several potential *daf-12* ligands, including previously known Δ4- and Δ7-dafachronic acid. Thereby it has been found that Δ1,7-dafachronic acid is the most abundant ligand in WT worms [59].

Nicotinamidase deficiency slows reproductive development in *C. elegans*. Wang et al. used UPLC-MS and GC-MS to find metabolic changes in *pnc-1* mutants. Using targeted analyses, they found decreases of NAD+ and glycolytic intermediates suggesting that efficient glycolysis seems to be mandatory for reproductive development [74].

### 3.2. Ascarosides and Other Signaling Molecules

**Figure 1:**
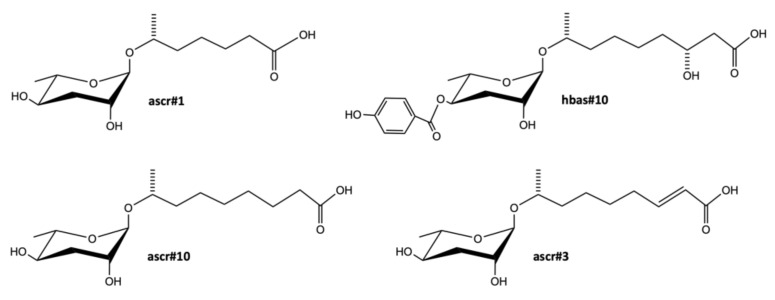
Example of ascaroside-structures: ascr#1, hbas#10, ascr#10 and ascr#3.

Individual *C. elegans* communicate with each other using a blend of small molecules excreted into the environment. This small molecule signaling has high relevance for the development and the behavior of the nematode. These molecules are called ascarosides and are chemically defined as glycosides of ascarylose, a dideoxysugar. They contain a hydrophobic tail derived from long and very long chain fatty acids and can contain several different, additional modifications at various positions, as shown in Figure 1. Ascarosides are involved in dauer formation, male attraction and hermaphrodite repulsion and aggregation.

Srinivasan et al. used activity guided fractionation, 2D-NMR and targeted LC-MS measurements to identify mating signals, which were excreted by L4, YA and adult hermaphrodites. Three ascarosides called ascr#2, ascr#3 and ascr#4 were identified and the attraction seemed to be concentration dependent. While males were attracted at low concentrations, deterrence occurred at higher concentrations [49]. Further investigations using differential analysis by 2D-NMR in combination with LC-MS led to discovery of previously unknown ascaroside species called ascr#6.1, ascr#6.2, ascr#7 and ascr#8 [26]. It has been shown that DANS is well suitable for the discovery of ascarosides since it can resolve them from chemical background in the spent cultivation media such as amino acids, peptides and other compounds which otherwise dominate the total ion chromatogram (TIC) at LC-MS. On the other hand, LC-MS was more beneficial for the detection of low concentrated ascarosides as ascr#1, ascr#6.1 and ascr#6.2.

Targeted analysis using LC-MS was used to profile differences in the ascaroside production between males and hermaphrodites. Hermaphrodites mainly produced ascr#3, containing an α,β-unsaturated fatty acid, while the saturated version ascr#10 was more dominant in males. Interestingly, this slight structural difference, shown in Figure 1, significantly affects signaling properties. ascr#3 deters hermaphrodites and attracts males while ascr#10 strongly attracts hermaphrodites [24]. Moreover, Srinivasan et al. found that indole ascarosides as icas#1, icas#3 and icas#9 attract both, males and hermaphrodites at high concentrations, whereas at low concentrations males were no longer attracted [122].

Comparative metabolomics by detailed analysis using LC-MS/MS with positive and negative ionization of different mutants of peroxisomal β-oxidation detected in total 146 ascarosides of which 124 were previously unreported. This allowed to draft a metabolic pathway of ascaroside biosynthesis and showed that the assembly of ascarosides includes building blocks from carbohydrate, lipid, and amino acid metabolism. The different phenotypic output of signaling of dauer formation, male attraction, hermaphrodite repulsion or aggregation were linked to the diversity of ascarosides [27].

GC-MS with EI has been evaluated as additional tool for analysis of ascarosides. Also, high resolution and peak shape for highly polar short chain components as ascr#5 were obtained, which are poorly retained by RPLC. Furthermore, identification can be achieved by a characteristic combination of fragments derived from the ascarylose. In contrast to LC-MS, GC-MS enables analysis of all basic ascarosides, but failed to detect any of the more complex ascarosides such as indole modified ascarosides [28].

### 3.3. Lipid Metabolism and Regulation

Regulation of fatty acid and lipid metabolism has been extensively studied in *C. elegans*. Additional techniques different to analysis methods for polar metabolites are necessary due to the very different physicochemical properties of lipids. Different possibilities for analysis of fatty acids and lipids exist and have been applied to *C. elegans*. GC-FID or GC-MS are most widely used for analysis of fatty acids, but also sterols, while intact lipids are analyzed by means of shotgun or LC-MS based lipidomics. Methods for the analysis of lipids and lipid metabolism are reviewed elsewhere [123]. 

**Figure 2:**
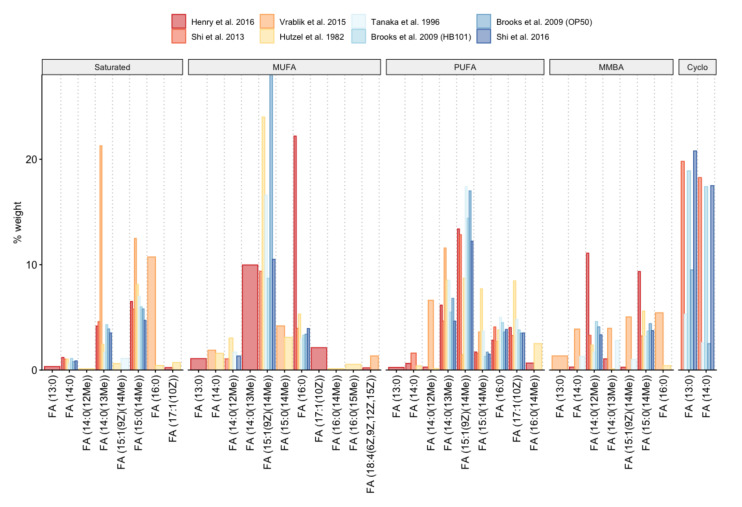
FA profiles from different publications. From left to right: saturated, monounsaturated (MUFA), polyunsaturated (PUFA), monomethyl branched chain (MMBA), and Cyclopropane (Cyclo) fatty acids. Publications that were included: Henry et al., 2016 [75], Shi et al., 2013 [90], Vrablik et al., 2015 [93], Hutzel et al., 1982 [125], Tanaka et al., 1996 [126], Brooks et al., 2009 (HB101 & OP50) [127], and Shi et al., 2016 [128].

The genetic basis for the biosynthesis of fatty acids have been unraveled by Watts and Browse using GC-FID [124]. The fatty acid composition of *C. elegans* and individual lipid classes has been determined multiple times [42,75,85,90,93,125,126,127,128]. A comprehensive overview of the *C. elegans* lipidome can be found in the review by Witting et al. [123]. Figure 2 illustrates the percentage of certain FAs that have been determined in several publications. One of the more recent investigations was performed by Henry et al., which determined fatty acid composition of WT worms using EI and CI at GC-QToF-MS. 28 fatty acids have been identified using their accurate mass and isotopic ratio, obtained at CI, and accurate mass fragmentation pattern provided by EI [75]. 

Although the actual fatty acid content is very dependent on the actual bacterial diet [127], several key features are conserved from different analyses. Several fatty acids are directly taken up from the food bacteria, while others are synthesized entirely by *C. elegans*. While the *E*. *coli* diet of *C. elegans* is rich in the saturated fatty acids lauric acid, myristic acid and palmitic acid, they are only minor species in *C. elegans*. Interestingly, palmitic acid and stearic acid have high percentages in Phosphatidylinositols (PIs) [85]. The nutritional cyclopropane fatty acids cis-9,10-methylene hexadecanoic acid (FA (17:0Δ)) and cis-11,12-methylene octadecanoic acid (FA (19:0Δ)) are enriched in triacylglycerols compared to other lipid classes, but are also found to a certain extent in other lipid classes. Some differences between lipid classes in their fatty acid composition exist. PC and PE are the major building blocks of membranes. Branched chain fatty acids 13-Methylmyristic acid (FA (14:0(13Me)) and 15-Methylpalmitic acid (FA (16:0(15Me)) are exclusively produced by *C. elegans* [89]. However, in most fatty acid profiles they were only found as minor species.

The *C. elegans* lipidome consists of several different lipid classes and shows several parallels to other lipidomes but has also several particularities. A particular example are sphingolipids, which are specifically found in *C. elegans* and makes it different from mammalian lipid metabolic pathways. In *C. elegans* they contain an unusual, branched chain sphingoid base derived from condensation of 13-methyl myristic acid with serine, resulting in 14-methylhexadecasphinganine [39,100,129,130]. Different sphingolipids were targeted in several investigations. For example, Mosbech et al. found that mutation of different ceramide synthases has different effects on lifespan. While loss of HYL-2, required for the synthesis of shorted chain sphingolipids with N-acyls smaller than 22 carbons, decreases lifespan, loss of HYL-1 and LAGR-1 is not affecting the lifespan [77]. Likewise, Menuz et al. found that loss of function of HYL-1 leads to increased resistance to anoxia [86]. Recently, an in-depth investigation to define the *C. elegans* sphingolipidome has been performed. Extensive fractionation of lipid extract together with UPLC-UHR-ToF-MS based analysis was used to identify lipids from different classes of sphingolipids [39]. Furthermore, *C. elegans* contains an interesting class of modified glucosyl-ceramides, which have an unusual PE or mono-methyl-PE modification on the sugar residue. These molecules have been shown to be able to rescue phenotypes derived from sterol-depletion [131]. An interesting approach for more detailed structural elucidation of SM species in *C. elegans* was developed by Zhao et al. Fragmentation of the [M+HCO_3_]^−^ adduct of SM led to radical-directed dissociation, yielding a high abundant intact N-acyl-chain fragment allowing to determine the composition of the sphingoid base and N-acyls [132].

The fatty acid profile of sphingolipids shows a different fatty acid profile compared to glycero- and glycerophospholipids. Typically, a mild alkaline saponification is used to generate free fatty acids from glycero- and glycerophospholipids, which are in turn analyzed as their methyl esters by GC-MS. Amide bound fatty acyls in sphingolipids are inert under this condition and require a different strategy. Therefore, total fatty acid profiles normally represent ester bound fatty acids ignoring fatty acyls from sphingolipids. Chitwood et al. and Gerdt et al. performed analysis of glucosylceramides in *C. elegans* and identified several 2-hydroxy fatty acids, which are not found in glycero- and glycerophospholipids. These fatty acids represent long chain saturated fatty acids ranging from 16 to 26 carbons and also include odd numbered chains. Interestingly, also even numbered iso branched chain fatty acids were reported [133,134].

Another *C. elegans* specific lipid class has been identified, investigating the lipidome of dauer larvae. The new lipid class, named maradolipids, are chemically 6,6′-di-*O*-acyltrehaloses with specific fatty acid composition and has been identified by 2D-NMR and analyzed by shotgun lipidomics. Maradolipids might be important for understanding the chemical basis for the resistance of dauer larvae to extreme environmental stress [30]. Further analysis of the *C. elegans* lipidome by shotgun lipidomics led to the discovery of lysomaradolipids [87]. Recently, analysis of maradolipids from dauer larvae using LC-IM-MS has been performed. DIA was combined with IM separation to obtain fragmentation data of maradolipids, which led to the identification of 45 marado- and lysomaradolipids directly from dauer larvae lipid extracts, without the need of further prior purification of glycolipids [135].

Ether lipids are an important part of the lipidome of *C. elegans*. Interestingly, in contrast to mammals, it contains mostly ether-linked phosphatidylethanolamines. Plasmenyl-PE (P-PE) and Plasmanyl-PE (O-PE) represented 5.1% and 4.9% of phospholipid content as determined by LC-MS/MS. These lipids mostly contained a 18:0 side chain at the sn1 position linked as ether or vinyl-ether [136]. Upon mutation of key enzymatic genes of ether lipid biosynthesis an increase in *de novo* fatty acid biosynthesis and reduction in the desaturases *fat-5* and *fat-7* was observed, which indicates a response to altered lipid composition upon ether-lipid deficiency [128].

Dietary restriction (DR) also has profound effects on the lipid metabolism and lipid levels. Klapper et al. studied supplementation with choline during DR and has seen that lipid stores are utilized at a later time point. Interestingly, choline supplementation only changed phenotypes related to lipids, but did not reverse the increase in lifespan [38]. *C. elegans* stores excessive energy in lipid droplets consisting of triacylglycerols. Although believed to be rather “inert” and only utilized open energy demand, they can be indicative of changes in lipid metabolism and serve as a buffer for fatty acids not used. Therefore, studying lipid storage is important to understand lipid metabolism. Schmökel et al. performed analysis of genetic of lipid storage in *C. elegans* embryos. Different genes were screened for a large lipid droplet phenotype. An interesting hit was *asm-3*, which is a member of acid sphingomyelinase genes. The closely related genes *asm-1* and *asm-2* did not show the large lipid droplet phenotype, suggesting a specific role for *asm-3*. Using lipidomics by LC-MS/MS difference in several lipid species were observed, while the total lipid content and the relative class distribution did not change too much [78].

The synthesis of lipids requires coordination of different genes in different metabolic pathways. The transcription factor SREBP-1 (sterol regulatory element binding protein) is one of the central transcription factors of lipogenesis. Low levels of PCs stimulate SBP-1/SREBP-1. Using a targeted RNAi screen Smulan et al. identified *lpin-1* and *arf-1*.*2* to be important for low-PC activation of SBP-1/SREBP-1 [137].

Several lipid related genes are still orphan in regard to their exact function. *lipl-5*, a lipase-like gene, is regulated by nutritional status, with starvation increased expression of *lipl-5*. It has been shown that mostly ceramides and mitochondrial lipids such as cardiolipins are affected by *lipl-5* mutation. Differences in cardiolipins were accompanied by differences in mitochondrial activity [94].

Recent publications show that a strong link between mitochondrial activity, longevity and lipid metabolism exists. Haeussler et al. studied mitochondrial dynamics in *C. elegans* and found that autophagy compensates for defects in mitochondrial fusion. Mutation in *fzo-1* led to fragmented mitochondria, decreased mitochondrial membrane potential and induced the mitochondrial unfolded protein response. Comparing the levels of TG species between wildtype N2 and *fzo-1* mutants, species with less carbons and double bonds were down-regulated, while longer and more unsaturated ones were up-regulated [95]. Mitochondrial dynamics and mitochondrial translation are linked to longevity via the transcription factor HLH-30 [138]. *hlh-30* mutants are susceptible to starvation. WT worms showed increased long-chain acyl-carnitines while short-chain acyl-carnitines were mostly unchanged. In the lipidomics analysis cardiolipins were found to show a similar pattern. Upon mutation of *hlh-30* this effect was abolished. Further analysis showed that *hlh-30* mutants shift their metabolism from mitochondrial to peroxisomal beta-oxidation. Further knockdown of *prx-5*, compromising the generation of peroxisomes, further rendered *hlh-30* mutants hypersensitive to starvation [96].

Lipid analysis has also been performed in the context of drugs slowing down ageing. Admasu et al. used a combination of different drugs extending the lifespan of *C. elegans*. One of the tested drug combinations required master lipid regulator transcription factor *sbp-1* to be present. Changes in desaturation of lipids has been linked to the extended life span, with a higher monounsaturated fatty acids (MUFA) to polyunsaturated fatty acids (PUFA) ratio. PC, PE and TGs lipids have been detected using LC-MS [97,98]. 

Gao et al. not only used analysis of polar metabolites as described above, but also performed analysis of lipids over a period of ten days or under different feeding conditions. In contrast to many other studies, which used RP based lipid analysis, they employed a normal phase separation. They determined changes in fatty acids and phospholipids in *mdt-15* mutants. *mdt-15* controls the expression of FA desaturase genes as *fat-7* and is therefore required for the synthesis of PUFAs. They found increased C18:0 and decreased C18:1 and PUFAs such as C18:2, C20:3, C20:4 and C20:5 levels in *mdt-15* mutants. Also PL having saturated acyl chains showed higher levels and PLs containing PUFAs, such as PS(38:7), PI(40:9), or PC(40:10), were decreased in *mdt-15* deficient worms [84].

Changes in the lipid metabolism of dauer larvae has been studied by Lam et al. using HPLC-MS/MS. They found that membrane phospholipids in dauer larvae are enriched in PUFAs. This enrichment is accompanied by an increase of free PUFAs as well as derived oxidative metabolites, such as prostaglandins. Release of PUFAs bound in membranes and higher levels of eicosanoid metabolites are hallmarks of termination of the dauer stage [99].

Metabolomics and lipidomics are also useful to elucidate the function of unknown genes. Schwudke et al. used top down lipidomics to elucidate the impact of the knockdown of *pmt-1* and *pmt-2* on the worm lipidome. Analysis of fragmentation data identified monomethyl-phosphatidylethanolamine (MMPE) and dimethyl-phosphatidylethanolamine (DMPE) as intermediates of PC biosynthesis from PE. *pmt-1* and *pmt-2* are homologues to plant methyltransferase. *pmt-1* deficient worms arrest their development at the L4/early adult stage and *pmt-2* worms at L3 stage. Upon silencing of *pmt-1* a decrease in MMPE and DMPE was observed, while RNAi of *pmt-2* lead to an increase of only MMPE [71].

Insights gained into lipid metabolism so far represented snapshots of the current state of lipid metabolism. However, metabolism is a dynamic phenomenon and changes in lipid levels might have been achieved by different means of re-routing fluxes. Isotopic labeling can be used to follow different tracer substances and their metabolism. A first example in *C. elegans* applied to lipid analysis has been described by Perez and Van Gilst as already described above. Fatty acids have been analyzed by GC-MS. If either non-labeled or completely labeled fatty acids were detected, they have been directly derived from the maternal fatty acid pool or the diet. Since *C. elegans* uses dietary metabolites for *de novo* biosynthesis, fatty acids that have been produced by the worm will show a mixed labeling pattern. Using this approach, it was found that >99% of mmBCFAs in *C. elegans* are *de novo* synthesized while saturated fatty acids are mostly taken directly up from the diet. Unsaturated fatty acids showed mixed synthesis rates. However, only fatty acids until 18 carbons were examined, since longer PUFAs show too much fragmentation and overlapping isotopic patterns, which could not be deconvoluted. Furthermore, differences in fatty lipogenesis between different alleles of *daf-2* mutation were studied [89]. The same approach was used to further study membrane dynamics. Comparing the percentage of newly incorporated fatty acids into either phospholipids or neutral lipids it was shown that phospholipids have a higher turnover rate. However, the amount of fatty acids from *de novo* synthesis was only for palmitic acid significantly different. Turnover rates were determined in different mutants and identified *fat-5*, *fat-6* and *fat-8* as membrane maintenance regulators. Furthermore, ^15^N tracers were used to follow the phospholipid turnover in more detail. This analysis revealed that *fat-7* also influences the turnover of lipids towards slower metabolism [139].

Another sub-category in lipidomics is steroid analysis, or steroidomics. The currently best known steroid pathways are related to dafachronic acids [140]. They play an important role as signaling molecules in dietary restricted worms and Dauer formation [141]. There are several potential *daf-12* ligands, as Δ4- and Δ7-dafachronic acid. Due to activity guided fractionation Δ7- and Δ1,7-dafachronic acid have been identified as most abundant ligands in *daf-12* mutants [59]. Different methods for quantification of dafachronic acid have been reviewed elsewhere [29]. Recently, the loss of *sul-2*, the steroid sulfatase has been investigated in *C. elegans*. Steroid hormone sulfatases are involved in development of hormone-regulated cancers, highlighting the importance of their investigation. Researchers found increased longevity in *sul-2* mutants and also sulfated hormones have been increased compared to WT worms [142].

### 3.4. Food Source and Nutrition

Changes in the *C. elegans* metabolome are depending on the food source and nutritional status [40]. Worms fed with different bacteria by show differences in their fatty acid composition. Mainly saturated FAs were directly taken from the diet and unsaturated FA were mixed. For instance, higher abundance of C15:0 and C17:0 fatty acids were observed when fed with *Bacillus subtilis* instead of *E*. *coli*. Thereby, most fatty acids of the worm reflect the fatty acid composition of the bacteria they eat, while some other fatty acids were absent in all tested bacterial strains. These fatty acids were hence mainly synthesized *de novo* in *C. elegans*. However, changes in lipid metabolism strongly depends on different alleles [42,89]. On the other hand, very different bacterial amino acid profiles lead to almost identical worm amino acid composition. This suggests that amino acid levels were tightly controlled and regulated in *C. elegans*. 

### 3.5. Other Topics

Beside the above-mentioned topics, several other investigations have been performed, e.g., toxicity exposure have been accomplished using *C. elegans* as a model. For example, cadmium exposure has been found to decrease cystathionine levels and increase phytochelatin levels due to upregulated methionine transsulfuration pathways, suggesting that cadmium is excreted as a phytochelatin-bound complex as a detoxification mechanism [143]. Exposure to nickel and the pesticide chlorpyrifos affected both mostly branched chain amino acids, lactate, and other energy generation related metabolites [144]. Also, phosphine exposure was investigated in WT and *dld-1* mutants. *dld-1* mutants were less affected by phosphine, whereas WT worms showed an accumulation of branched-chain amino acids, glutamate, and glycine [48]. Lastly, Sudama et al. investigate changes of metabolites due to lead exposure. They used HPLC with coulometric array detection to detect and quantify metabolites based on their oxidation-reduction potentials. After lead exposure, changes in tryptophan, tyrosine and purine have been observed in WT worms [145]. 

The small nematode may also be used as a model for human diseases as shown by Van Assche et al., who were interested in Alzheimer disease (AD). They studied changes in the metabolism after induced expression of human amyloid-beta peptide by GC-MS, RPLC-MS and ANP-LC-MS, which represents an alternative for the separation of polar substances. ANP columns show only small interactions with water, avoiding the major disadvantage of classical NP [146]. Results confirmed previous observations from experiments in AD, also in humans, as for instance increased levels of allantoin [79]. Moreover, Teo et al. combined metabolomics data with transcriptomics and computational modeling, and firstly linked amyloid-beta expression to TCA cycle [31]. 

AD but also Parkinson’s disease are neurodegenerative-related diseases believed to be caused by oxidative stress, among other things [147,148,149]. Helmer et al. recently investigated the role of cardiolipins (CLs) as oxidative stress marker in *C. elegans*. Oxidative stress was induced by supplementation of *tert*-butyl hydroperoxide (tBOOH), resulting in oxidized CL species. CL analysis was performed using 2D-LC-HRMS in order to remove co-eluting lipid species. They identified oxidation products of CLs such as CL 80:14, CL 80:15 and CL 80:16 most abundantly in stressed worms. Based on their high content of poly-unsaturated fatty acids, CLs have been shown to be suitable biomarkers for oxidative stress [150]. 

Because of its fast development, *C. elegans* is also an ideal organism for mutation accumulation (MA) experiments. MA experiments give insights about the genetic variation originating from spontaneous mutations. Herby, mutations accumulate in genomes for many generations. The majority of mutations is however expected to be fixed randomly. Therefore, new mutational variances per generation are determined [151]. GC-MS based metabolomic analyses identified 29 metabolites that vary greatly in their vulnerability to mutation [91].

Also, cell death has been investigated in *C. elegans* using 2D-NMR based comparative metabolomics. Researchers identified a blue fluorescent substance that accumulates at *C. elegans* death as anthranilic acid glucosyl esters derived from tryptophan [152].

## 4. Publicly Available Datasets

Since *C. elegans* represents an interesting and important model organism, the availability of public data is an important issue. On the one side for further development of bioinformatics approaches and on the other side for the reconstruction of the *C. elegans* metabolome and lipidome. There are also certain publicly available datasets of metabolomics studies of *C. elegans* from different analysis types. Up to date, there have been nine metabolomics studies published on Metabolomics Workbench (https://www.metabolomicsworkbench.org/) and four studies on MetaboLights (https://www.ebi.ac.uk/metabolights/). These contain various information such as sample details, sample-preparation protocols, analyses, post-processing details or annotations. Public deposition of further datasets would facilitate the reconstruction of the *C. elegans* metabolome, allows the extraction of spectral information for secondary metabolites, for which no commercial reference standards are available and extraction of reference values or even concentrations for future use.

## 5. Prototyping the *C. elegans* Metabolome

Since currently no *C. elegans* metabolome and/or lipidome database exists, we aimed to collect metabolites and lipids present in *C. elegans*. While reviewing articles on *C. elegans* metabolomics and lipidomics, we have curated metabolites and lipids that have been detected so far in *C. elegans*. Metabolite names were curated from the main text, figures and tables as well as potential supporting information. Corresponding data is collected in a central GitHub repository (https://github.com/wormjam-consortium/wormjam-db) and freely available. 

The data is grouped into two libraries. The first library represents metabolites predicted to be present in *C. elegans* based on the WormJam metabolic model. We extracted metabolites from the current version of WormJam v0.1.0 (https://zenodo.org/record/3978712#.X47IqlngpmB, https://github.com/wormjam-consortium/wormjam/releases). The WormJam metabolite library contains in total 1215 unique metabolites, of which 1205 have a structure associated with it. However, this library is purely in silico. 

**Figure 3:**
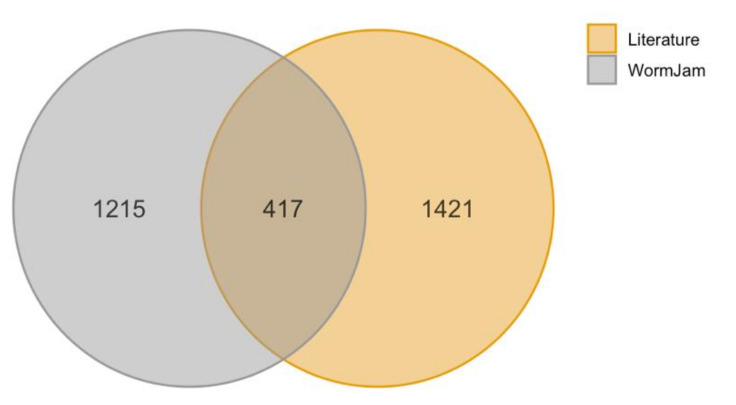
Venn diagram of overlap of metabolites between literature (yellow) and WormJam (grey) based on the first block of their InChIKey.

The second library is based on the literature curation. Since this library might contain different versions of the same metabolite, e.g., full stereochemistry vs. generic structures (e.g., L-glycine vs. glycine) or ions vs. neutral molecules (e.g., lactate vs. lactic acid), we tried harmonizing all names. Besides the correct chemical name, we additionally curated the chemical formula, InChI, InChIKey, SMILES, ChEBI, KEGG, BioCyc, HMDB, LipidMaps, SwissLipids, Wikidata, PubChem, Metabolights and ChemSpider IDs for all metabolites in both libraries, where available. Different IDs were retrieved using BridgeDB [153]. In total 1421 unique metabolites have been curated from literature. We compared predicted and detected metabolites in *C. elegans* based on the first block of their InChIKey (if available) and found that only 417 metabolites were overlapping, while further 1025 have been detected so far but are not found in the WormJam model (Figure 3). The model contains 819 further metabolites that have not been detected yet. Metabolites from literature and from the WormJam model have been combined afterwards to a single consensus database, representing a prototype database of the *C. elegans* metabolome. 

In order to obtain an overview on the properties of the *C. elegans* metabolome and methods that might be required to fully capture it, we combined the list of predicted and curated metabolites into a single database. To estimate a proper analytical platform, we need to gain more information about the molecular properties of the metabolites. For this purpose, we used the rcdk package in R to calculate various physicochemical properties of the metabolites, based on their SMILES [154]. 

**Figure 4:**
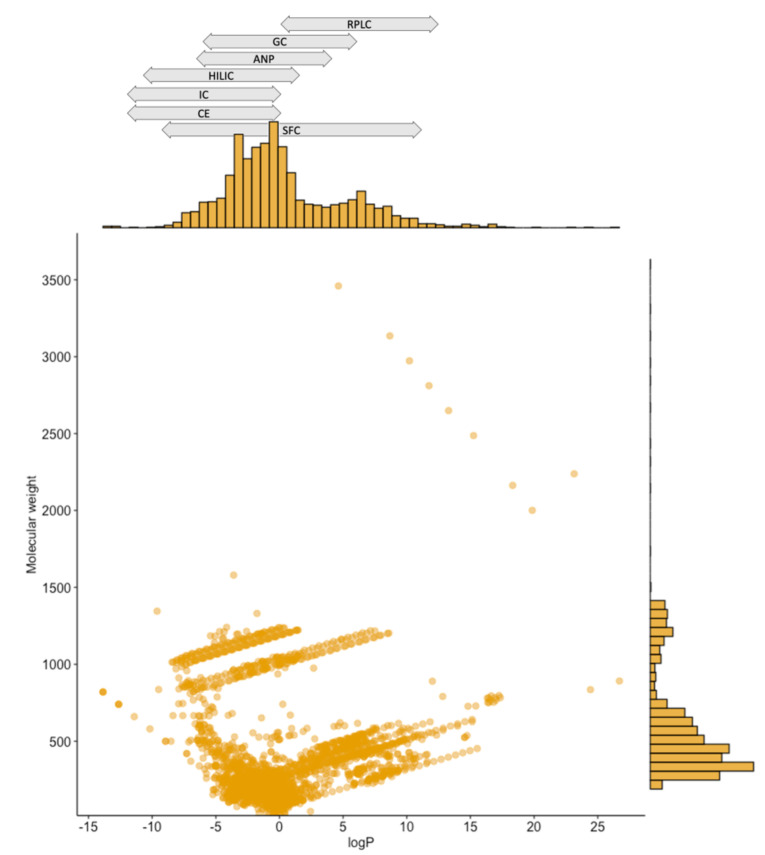
Molecular weight and logP distribution of merged molecules from the WormJam model and curated from literature. Different separation methods and their polarity are displayed in the logP range. Reversed phase liquid chromatography (RPLC), gas chromatography (GC), aqueous normal phase (ANP) chromatography, hydrophilic interaction liquid chromatography (HILIC), ion chromatography, capillary electrophoresis (CE) and supercritical fluid chromatography (SFC).

Figure 4 shows the molecular weight (MW) and logP distributions of the metabolites combined from literature and WormJam. The logP value is a measure for the hydrophobicity/hydrophilicity of a molecule. It is defined by the partition coefficient P which is basically the solubility of a molecule between an octanol-water phase. The majority of metabolites (74%) have a lower mass than 500 Da. On the contrary, 17% of the metabolites are above 800 Da. The majority (58%) of metabolites from the *C. elegans* prototype metabolome database is very polar, having logP values below 0. And 9% of the metabolites are having logP values below −5. On the other hand, there are 19% of very lipophilic metabolites, having logP values above 5. In particular, metabolites in the higher mass-range are originally metabolites from the model, which are mainly coenzyme A (CoA) derivatives. Detected lipids curated from the literature were not counted in, since no definitive structure in form of a SMILES was given for them (see below).

In order to cover (more) highly polar metabolites analytically, more techniques specialized in this polarity range needs to be applied. Figure 4 also displays the polarity of different analytical separation methods in the logP range. Separation methods as SFC, RPLC, GC, and ANP-LC are more suitable for mid- up to non-polar compounds. Promising separation techniques for highly polar substances are especially HILIC, ion chromatography (IC), and CE. Since very high salt concentrations are used in IC, it is less suitable for MS coupling. Therefore, we suggest investigating the *C. elegans* metabolome in more detail using HILIC-MS and CE-MS. 

The separation mechanism in HILIC is more based on interactions of the molecules with the stationary phase and the water layer on the stationary phase surface respectively. In contrast, in CE molecules are separated due to different mobilities in an electric field. Besides polarity, the main requirement for separation in CE is charge, which is not necessary in HILIC. Since the separation mechanism between CE and HILIC is different, the two would offer different possibilities and selectivity’s for separation of polar metabolites. 

To demonstrate whether the *C. elegans* metabolome contains suitable CE candidates we determined the proportion of molecules having a permanent or pH-dependent charge. 157 structures of the combined database were associated with a permanent charge, based on their SMILES. However, all molecules except of 11 have an ionizable group and thus can be made applicable for CE separation. Since CE-MS has not yet been used in *C. elegans* metabolomics, we strongly believe that this technique allows taking further steps in unravelling the *C. elegans* metabolome. 

Beside metabolites, several different lipids classes and species have been detected. Identifications were reported on different levels of detail in regard to the structural information. Analysis of lipids has been performed with different approaches. Likewise, lipids were reported on different levels of detail. In order to generate a harmonized list of detected lipids the naming has been standardized on the lipid species level. Since no explicit structure is available for lipids species, no InChI, InChKey or SMILES are available. In order to be able to compile this into a single list of *C. elegans* lipids they have been all normalized to the lipid species level according to Liebisch et al. [155]. In total 1402 unique lipid species have been curated together with their molecular formula. The list of unique metabolites and lipids are available from GitHub. We welcome future suggestions for publications to be added to the collection as well as further collaboration in the curation process. 

## 6. Conclusions and Outlook

In this review, we provided an overview about recently used metabolomics analytical platforms and biological applications from the model organism *Caenorhabditis elegans*. *C. elegans* is an ideal organism for metabolomic investigations because of its invariant cell number, easy and fast cultivation and its short lifespan. One of the most common applications in the field of metabolomics are studies of long-lived mutants and ageing in the worm. Thereby, the most studied long-lived mutant is *daf-2*. Similar to other long-lived mutants, as for instance *eat-2*, *slcf-1*, *ife-2,* but also dauer larvae, they share a metabolic signature of longevity as for instance changes in amino acids, carbohydrate, choline or upregulated lipid storage. However, it still remains elusive, which changes in metabolism corroborate long live and which are not related. Metabolomics studies have predominantly been performed using, either individually or in combination, analytical platforms as 1D- and 2D-NMR approaches, and GC and LC with different column selectivity coupled to MS. As the properties are very different to metabolites, lipids are mostly studied individually with their own methods. They are predominantly analyzed by mass-spectrometry, either at direct-infusion or after chromatographic separation. 

The *C. elegans* metabolome and lipidome covers a large range of polarity, so multiple analytical methods need to be combined to achieve true comprehensive profiling. Especially, the highly polar region (negative logP values) requires further developments.

Other promising analytical separation techniques prior MS analysis that have not yet been applied to *C. elegans*, but in other metabolomics studies, are ion mobility or CE. IM may further enhance peak capacity of LC-MS data and might probably enhance annotation success of *C. elegans* metabolomics data. The benefits of using IM have been demonstrated in lipidomics, where it enables the detection of new species of maradolipids in the nematode. 

CE-MS is another rising powerful technique in metabolomics, which is especially suitable for highly polar and charged metabolites. Therefore, it is a complementary analytical platform to HILIC-MS. Due to its very different separation mechanism to LC, we strongly believe CE will help uncovering the *C. elegans* metabolome. Another characteristic in CE is the low injection volume, which is a few nl. Because of this, we think that CE-MS metabolomics analysis will require fewer amount of worms, compared to LC-MS. This would significantly reduce laboratory efforts and also minimize genetic variation. Given the above reasons, we see a great importance to expand these platforms in *C. elegans* metabolomics. 

Appropriate analytics, sample preparation and evaluations on metabolomics data needs to be adapted on the biological question. As generally valid for metabolomics, there is no analytical platform that provides a comprehensive analysis of the *C. elegans* metabolome. Bioinformatic analysis of (*C. elegans*) metabolomics data is also gaining increased importance. For example, pathway or network analysis such as GGM were increasingly applied. Flux balance analysis have also been shown as an effective tool to analyze metabolites from tissue specific pathways in *C. elegans*. There have been first steps in developing tissue and cell type specific models of the nematode. 

Lastly, we created a draft metabolomics/lipidomics database of *C. elegans* by curating all annotated metabolites from reviewed articles into a publicly available repository. Increasing this database and matching it to *in silico* databases as WormJam helps gaining new insights to the *C. elegans* metabolome and lipidome. Moreover, it may be useful to accelerate annotation of metabolomics data and avoid over annotation.

The remaining question is “quo vadis?”, where is *C. elegans* metabolomics and lipidomics heading towards? Current applications are mostly based on populations of *C. elegans* with different absolute number of worms per sample. However, although isogenic worms show an individual response to treatments, growth conditions, etc. Therefore, in future it will be important to go from populations to single worms. In contrast to DNA and RNA, proteins and metabolites cannot be amplified. This requires highly sensitive analytical workflows. A first example of single *C. elegans* proteomics has been demonstrated by Bensadeek et al. [156]. Likewise, a first application of single worm metabolomics using µHR-MAS-NMR has shown that metabolites from a single nematode is possible [62].

A further important step required is the creation of *C. elegans*-specific metabolite database following the example of HMDB [56], including metabolites, MS-, MS/MS- and NMR spectra. Potentially this can be realized under the umbrella of WormBase to provide a “one-stop-shop” for *C. elegans* scientists [157].

Since *C. elegans* represents a multicellular organism with differentiated tissues, the location of specific metabolites is an important factor. First tissue specific metabolic models have been developed [117]. The transparent body of *C. elegans* enabled many different microscopy methods to be used for localization of proteins, e.g., using GFP-fusions. Tissue specific metabolomics, e.g., using MS imaging shall be developed for the spatially re-solved metabolomics. Likewise, single-cell metabolomics generally gains interest and can be potentially applied to isolated *C. elegans* cells.

Although, it is not clear where the next developments will lead to, the future for *C. elegans* metabolomics and lipidomics is bright.

## Figures and Tables

**Table 1:** Overview on analytical methods used in *C. elegans* metabolomics/lipidomics and their advantages, disadvantages and corresponding references.

	Method	Advantage	Disadvantage	Remarks	References
NMR	^1^H NMR	Quantitative, non-destructive, minimal sample preparation	Only aqueous, high abundant metabolites	Overlapping signals result in ambiguity of metabolite identification	[20,30,40,41,42,43,44,45,46,47,48,49,50,51,80,81]
	DANS	Simple linking of metabolites with biological function	Only high abundant metabolites		[26,59]
	HR-MAS	No metabolite extraction needed, intact worms	Large populations of *C. elegans* needed, only high abundant metabolites		[60,61]
	HR-MACS + ^1^H NMR microprobe	Small number of worms can be analyzed	Only high abundant metabolites		[62,63]
	^13^C HMN + ^13^C-labeling	Much more metabolites detected than in ^1^H-1D NMR	Reduced sensitivity due to ^13^C-^13^C coupling, proper pulse program required (ct-HSQC)	Higher spectral range than at ^1^H NMR	[52,53,54,55,56,57,58]
MS	DI-MS	Fast and high throughput	Isomers cannot be differentiated	Frequently used in lipidomics	[30,71,82,83,84,85,86,87]
	GC-MS	High resolution, Absolute quantification possible	Derivatization necessary	Analysis of lipids and metabolites possible	[31,33,42,43,50,59,65,71,72,73,74,75,88,89,90,91]
	LC-MS	Absolute quantification possible, separation of isomers, a lot of metabolites may be identified	Lower resolution than GC	Selectivity depending on the stationary phase	[22,24,31,32,33,38,39,42,43,44,51,67,73,74,76,77,78,79,83,92,93,94,95,96,97,98,99,100]

## Data Availability

Curated metabolites and lipids were collected in a central GitHub repository (https://github.com/wormjam-consortium/wormjam-db) and freely available. All figures and tables of this manuscript are original.
